# Mitochondria, a Key Target in Amyotrophic Lateral Sclerosis Pathogenesis

**DOI:** 10.3390/genes14111981

**Published:** 2023-10-24

**Authors:** Emmanuelle C. Genin, Mélanie Abou-Ali, Véronique Paquis-Flucklinger

**Affiliations:** Institute for Research on Cancer and Aging, Nice (IRCAN), Université Côte d’Azur, Inserm U1081, CNRS UMR7284, Centre Hospitalier Universitaire (CHU) de Nice, 06200 Nice, France; melanie.abou-ali@etu.univ-cotedazur.fr (M.A.-A.); veronique.paquis@univ-cotedazur.fr (V.P.-F.)

**Keywords:** mitochondria, amyotrophic lateral sclerosis, motor neuron disease, frontotemporal dementia, ALS genes, *CHCHD10*

## Abstract

Mitochondrial dysfunction occurs in numerous neurodegenerative diseases, particularly amyotrophic lateral sclerosis (ALS), where it contributes to motor neuron (MN) death. Of all the factors involved in ALS, mitochondria have been considered as a major player, as secondary mitochondrial dysfunction has been found in various models and patients. Abnormal mitochondrial morphology, defects in mitochondrial dynamics, altered activities of respiratory chain enzymes and increased production of reactive oxygen species have been described. Moreover, the identification of *CHCHD10* variants in ALS patients was the first genetic evidence that a mitochondrial defect may be a primary cause of MN damage and directly links mitochondrial dysfunction to the pathogenesis of ALS. In this review, we focus on the role of mitochondria in ALS and highlight the pathogenic variants of ALS genes associated with impaired mitochondrial functions. The multiple pathways demonstrated in ALS pathogenesis suggest that all converge to a common endpoint leading to MN loss. This may explain the disappointing results obtained with treatments targeting a single pathological process. Fighting against mitochondrial dysfunction appears to be a promising avenue for developing combined therapies in the future.

## 1. Amyotrophic Lateral Sclerosis, a Motor Neuron Disease

Amyotrophic lateral sclerosis (ALS) is a fatal multisystemic and multifactorial motor neuron disease (MND) characterized by progressive degeneration of upper and lower motor neurons (MNs) in the cortex, brainstem and spinal cord [1,2]. The progressive loss of MNs causes severe muscle weakness and gradual paralysis, leading to death of patients by respiratory failure 3 to 5 years after the onset of symptoms [3].

ALS was first described by the French neurologist Jean-Martin Charcot in 1869 and is also known as Charcot disease or Lou Gehrig’s disease (from the name of a baseball player diagnosed with the disease). With an incidence of 1.4–1.8 cases per 100,000 individuals per year, ALS is the most common adult onset neurodegenerative disease after Alzheimer’s and Parkinson’s disease, respectively. However, the prevalence of ALS is 3.9–5.0 per 100,000 population, reflecting the rapid mortality of the disease [4]. The disease usually begins in late adulthood, but in rare cases it can occur in the juvenile stage (before 25 years of age) or at a young age (before 45 years of age). ALS incidence and prevalence increase with age until 70–79 years and are higher in men than in women (1.91 and 5.96 for incidence and prevalence in men and 1.36 and 3.90 for women, respectively) [4,5,6]. The cumulative lifetime risk of developing ALS is estimated at 1:350 for men and 1:400 for women [7]. Incidence is also higher in Europe and America than in Africa and Asia [5,8].

ALS has two typical clinical manifestations at disease onset, the spinal form and the bulbar form. The spinal onset of the disease occurs in about 70–75% of patients and begins in peripheral MNs. They first present limb involvement characterized by muscle weakness and wasting, starting distally or proximally in the lower and upper limbs. Gradually, spasticity develops in the limbs, affecting manual dexterity and gait, with cramps, fasciculation, tremors and muscular atrophy, leading to death within 3 to 5 years. The remaining patients (25–30% of cases) develop a bulbar onset of ALS, with the first symptoms affecting speech (dysarthria) and swallowing muscles (dysphagia). Limb symptoms may appear almost simultaneously or, in most cases, one to two years later. ALS bulbar form leads to patient death within 2 to 3 years of onset. Almost 85% of patients with the spinal form develop bulbar changes as the disease progresses [9].

The occurrence of ALS can be either familial (fALS), which accounts for 5–10% of ALS cases, or sporadic (sALS), which is the main cause. Hexanucleotide repeat expansion in the chromosome 9 open reading frame 72 (*C9ORF72*), superoxide dismutase 1 (*SOD1*), TAR DNA-binding protein 43 (*TARDBP*, *TDP-43*) and fused in sarcoma (*FUS*) are the most common causative genes, responsible for more than 50% of fALS and 7.5% of sALS. However, the remaining cases, particularly most sALS cases, are not due to variants of known causative genes. Although evidence is accumulating that genetic factors, environmental factors (i.e., heavy metal exposure, organic chemicals (pesticides, solvents, etc.)), a history of physical head trauma/injury and lifestyle [10]) and age-related factors play a potential role in the etiology of ALS. Their respective contribution or exact mechanism is still largely unknown. One of the hypothesis is that ALS development could be due to the coincidence of genetic predisposition and environmental exposure over time [11].

ALS overlaps clinically with several other adult-onset degenerative diseases, most commonly frontotemporal dementia (FTD). ALS and FTD are effectively part of the same clinical spectrum, with 50% of ALS patients showing frontal lobe involvement and 15% of FTD patients showing signs of ALS. The same genes are also involved in familial forms of these two diseases [12]. FTD is a complex and heterogeneous neurodegenerative disorder characterized by degeneration of the cortex of the frontal and antero-temporal lobes of the brain. Clinical manifestations are variable between patients and include significant personality and behavioral changes, as well as gradual impairment of the language skills and executive function. FTD is the second most common early-onset dementia in people under 60 years of age after Alzheimer’s disease, affecting approximately 3 per 100,000 people under 65 years of age annually [13]. ALS and FTD may share similar pathological phenotypes, particularly the accumulation of protein aggregates. Aggregation of TDP-43 proteins occurs in neurons and glia in around 97% of sALS cases and 40–50% of FTD patients [3,14,15]. To date, four major proteinopathies have been identified in FTD patients: tau, TDP-43, FUS and ubiquitin-positive inclusions [16,17].

As with other major neurodegenerative diseases, there is currently no cure or even effective treatment for ALS, FTD or ALS/FTD, hence the great interest in strategies aimed at identifying new therapeutic targets. Numerous cellular processes are damaged in ALS and ALS/FTD. These include impaired RNA metabolism, altered protein homeostasis with accumulation of aggregated proteins (TDP-43, FUS, SOD1…), impaired DNA repair, oligodendrocyte dysfunction and degeneration, neuroinflammation, defective vesicular transport, defective axonal transport, glutamate excitotoxicity, apoptosis, oxidative stress, endoplasmic reticulum (ER) damage and mitochondrial dysfunction [12,18,19,20].

The aim of this review is to provide an up-to-date overview of the many different physiological and pathophysiological roles of mitochondria in ALS pathogenesis.

## 2. Mitochondrial Dysfunction in ALS Pathogenesis

### 2.1. Mitochondria and ALS

Mitochondria are a double-membrane-bound organelle in most eukaryotic cells. They are composed of the outer mitochondrial membrane (OMM), the intermembrane space (IMS), the inner mitochondrial membrane (IMM) and the matrix. The main function of mitochondria is to generate ATP by oxidative phosphorylation (OXPHOS) of ADP via the electron transport chain. Mitochondria also play a major role in the maintenance of cellular homeostasis and apoptosis. Mitochondria are key regulators of calcium homeostasis, alone or in association with the ER. Physiological neuronal functions require a large amount of ATP and thus functioning mitochondria. Neuronal mitochondria are essential for neuronal function and survival.

Of all the factors involved in ALS, mitochondria have always been considered as a major player, as secondary mitochondrial dysfunction has been identified in various models and patients. Indeed, mitochondria are involved in cellular functions damaged in ALS such as calcium homeostasis, regulation of apoptosis and protein quality control [21]. Moreover, the identification of *CHCHD10* variants in ALS patients [22] was the first genetic evidence that a mitochondrial defect can cause MN damage and directly links mitochondrial dysfunction to the etiology of ALS [23,24] and FTD [25].

**Figure 1:**
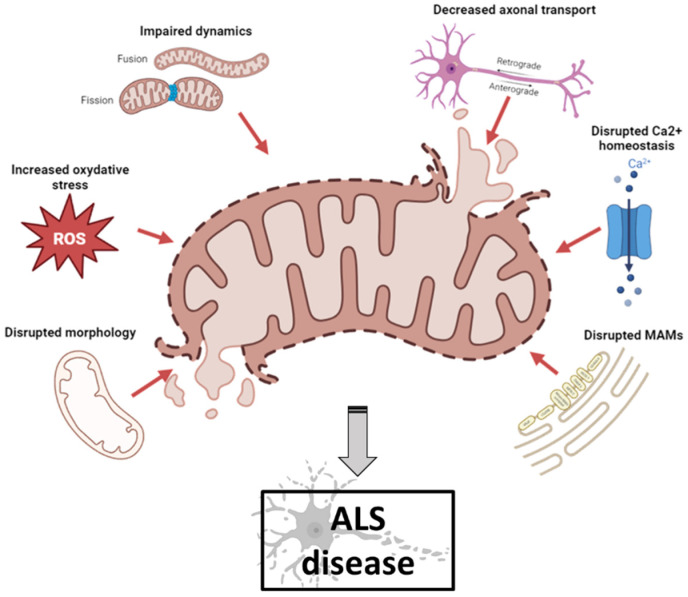
Schematic illustration of mitochondrial dysfunctions in ALS pathogenesis (created with biorender.com, accessed on 29 September 2023). Abnormal mitochondrial morphology, increased ROS production, defects in mitochondrial dynamics, impaired axonal transport, disruption of axonal transport and disruption of MAM integrity have been described both in ALS patients and in ALS models. ALS: amyotrophic lateral sclerosis; MAM: mitochondrial-associated membranes; ROS: reactive oxygen species.

Mitochondrial dysfunction occurs in numerous neurodegenerative diseases, particularly ALS, where it contributes to MN death [26]. Abnormal mitochondrial morphology, defects in mitochondrial dynamics, altered activities of respiratory chain enzymes and increased production of reactive oxygen species (ROS) have been described in both ALS patients and ALS models [27] (Figure 1).

### 2.2. Mitochondria and Ultrastructural Morphology

Mitochondrial morphology abnormalities and aggregated mitochondria were one of the first changes observed in ALS patients [28]. ALS MNs showed decreased mitochondrial membrane potential, impaired mitochondrial import, decreased OXPHOS and altered cristae [29,30]. Ultrastructural abnormalities of mitochondria (with vacuolated and swollen appearance, cristae abnormalities) have been associated with mitochondrial axonal transport damage. In a transgenic mouse model of fALS associated with SOD1 mutant, abnormal mitochondrial morphology and mitochondrial dysfunction in the nervous system occur early in disease progression, suggesting that these changes may predispose MNs to degeneration. Mitochondrial transport in neurons is progressively impaired early in the disease course before the onset of clinical symptoms, and retrograde transport is impaired earlier than anterograde transport [31].

### 2.3. Mitochondria and Reactive Oxidative Species (ROS)

In mitochondria, ROS are produced during physiological metabolism and are crucial in cellular homeostasis maintenance. Damage in OXPHOS results in a decrease in transmembrane potential and an increase in ROS production, leading directly to oxidative damage that causes mitochondrial dysfunction (generation of misfolded proteins and protein aggregates, etc.) and eventually mitophagy and apoptosis [32,33]. Oxidative stress is caused by an imbalance between oxidants and antioxidants resulting from an excess of ROS, reactive nitrogen species (RNS) or inadequate antioxidant system function. ROS contribute significantly to neuronal cell degeneration by modulating biomolecule function (DNA, RNA, lipids and proteins) and processes (nucleic acid oxidation, lipid peroxidation). Mitochondrial dysfunction and oxidative damage leading to MN degeneration have been extensively described in the pathogenesis of ALS [32]. Pathological mechanisms triggered directly or indirectly by ROS can lead to neuronal damage and degeneration [34]. MNs are extremely sensitive to oxidative stress and the central nervous system has low antioxidant capacity and low activity of protective enzymes, resulting in low cell regenerative capacity.

### 2.4. Mitochondria and Dynamics

Mitochondria form a dynamic network that is constantly dividing, fusing and changing size and shape. They constantly maintain a balance between two phenomena, fusion and fission, which are necessary for maintaining their integrity and quantity. These processes are essential for the proper functioning of these organelles. Mitochondrial fusion is the mechanism by which two mitochondria join together to form a new one. The exact event occurs by the fusion of the OMM and the IMM of the two mitochondria. Mitofusin 1 and 2 (MFN1 and MFN2) proteins are involved in fusion of the OMM, optic atrophy 1 (OPA1) protein mediates fusion of the IMM [35,36]. Mitochondria fission is the mechanism by which a mitochondrion is divided into two smaller mitochondria. This mechanism is mostly mediated by the dynamin-related protein (DRP1) and mitochondrial fission 1 (FIS1) protein. FIS1 localizes primarily on the OMM. DRP1 is a cytoplasmic protein that translocates to mitochondria and interacts with FIS1 to enhance fission. Fission takes place at ER–mitochondria junction sites and needs the oligomerization of DRP1 and several others proteins, such as FIS1 [37,38]. A positive mitochondrial membrane potential (120–200 mV) is fundamental for the physiological performance and survival of cells, especially those that have a high energy requirement. Thus, loss of mitochondrial potential membrane is an indicator of increased cell death. After fission, daughter mitochondria produced can either retain intact membrane potential or be depolarized. Depolarized mitochondria can then either return to a normal balance between fusion and fission or be eliminated by the specific autophagic process called mitophagy. Dysfunctional mitochondria show a fragmentation and perinuclear clustering, then turnover by mitophagy and degradation into lysosomes [39].

### 2.5. Mitochondria and Axonal Transport

Neurons are polarized cells composed of three distinct structural and functional domains: the cell body (soma), an elongated axon and the dendrites. Neurons are the longest cells and a highly structured transport machinery is needed to make them work properly. Axonal transport is essential for neuronal function, such as the maintaining of intracellular homeostasis and interaction between neighboring neurons. Transport within axons occurs in a bidirectional manner: the anterograde transport (cell body to distal axon) and the retrograde transport (distal axon to the cell body) [40]. Anterograde transport of newly synthesized mRNA and proteins, such as neurotransmitters, precursors and enzymes, to the distal axon terminal is required. It is mediated by kinesin movement along microtubules. Retrograde transport is necessary to maintain homeostasis by removing cytotoxic metabolites generated in the axon terminal and aged or damaged proteins and organelles targeted for degradation and recycling towards the neuron cell body [41]. Retrograde movement is mediated by dynein movement along microtubules.

Under physiological conditions, MNs are particularly dependent on mitochondrial ATP because of their high energy demand. Targeted transport of mitochondria from the neuronal soma to the periphery via axons is therefore essential for neuronal transmission through local ATP production. Mitochondrial dysfunction is one of the causes leading to axonal transport dysfunction. The transport and distribution of mitochondria in neurons is efficiently regulated in response to changes in neuronal activity and to a variety of physiological and pathological conditions. During axonal transport, mitochondria frequently change direction, pause or switch to permanent docking [42]. Specific mechanisms are necessary to transport mitochondria to their final destinations and to ensure that mitochondria remain stationary in regions of high energy demand and calcium homeostasis. In axon, mitochondria bind to motors by linking to their respective adaptor proteins, either directly or through mitochondrial receptors. Cytoplasmic dynein motors are responsible for retrograde transport of mitochondria to the soma, while KIF5 kinesin motors regulate the anterograde transport of mitochondria to distal axonal regions and synaptic terminals. Mitochondrial axonal transport is often impaired in ALS.

### 2.6. Mitochondria and Calcium Homeostasis

Mitochondria are critical for calcium homeostasis, and calcium is needed for normal transmission of signals between neurons, notably to modulate neurotransmitter release in neuromuscular junctions (NMJs). Mitochondria regulate cellular calcium ions (Ca^2+^) by sequestering and releasing Ca^2+^. Mitochondrial Ca^2+^ is also crucial in production of ATP, regulation of mitochondrial metabolism and cell death. Abnormalities in cellular Ca^2+^ signaling are common features in the pathogenesis of ALS.

Mitochondria regulate Ca^2+^ concentration through voltage-dependent anion-selective channel proteins (VDAC), which move Ca^2+^ from the matrix into the mitochondrial intermembrane space via the Ca^2+^ uniporter complex in the matrix [43]. Ca^2+^ release occurs at the apposition of ER and mitochondrial membranes, termed mitochondrial-associated membranes (MAMs) [44]. These structures control the entry of Ca^2+^ into the matrix of the mitochondria [45]. The main effectors of the Ca^2+^ release pathway in the ER are the inositol 1,4,5-triphosphate receptors (IP_3_R) and ligand-gated channels activated by the second messenger IP3 produced in response to several different extracellular signals [46]. The IP_3_R/VDAC-complex is one of the most important protein assemblies linking the ER and mitochondria [47,48]. The IP_3_R releases Ca^2+^ from the ER, and VDAC1 is responsible for Ca^2+^ transfer across the OMM. The interaction between the ER protein and the vesicle-associated membrane-protein-associated protein B (VAPB) and the OMM protein, protein tyrosine phosphatase interacting protein-51 (PTPIP51), facilitates IP_3_R-mediated Ca^2+^ transfer from ER to mitochondria, mitochondrial ATP production and autophagy [49,50]. Controlled Ca^2+^ release leads to the activation of OXPHOS activity and ATP production. When Ca^2+^ release is disrupted (continuous or excessive release), mitochondrial Ca^2+^ overload, opening of the mitochondrial permeability transition pore and triggering of the intrinsic apoptotic program are observed [51,52].

### 2.7. Mitochondria and MAM

The contact sites between the mitochondria and ER, called MAMs [53], are crucial for the maintenance of cellular homeostasis, including energy metabolism, Ca^2+^ homeostasis, cholesterol and phospholipid synthesis, ER stress response, autophagy, inflammation and mitochondrial biogenesis and transport [20,54]. MAM integrity is generally compromised in ALS [18] and in other neurodegenerative disorders, such as Alzheimer’s disease [55], Parkinson’s disease [56] and Huntington’s disease [57]. Moreover, MAM integrity is essential for mitochondrial dynamics (i.e., fission and fusion). Various proteins associated with mitochondrial dynamics are accumulated in the MAMs, such as mitofusin 2 (MFN2), interacting with apoptosis regulator protein bax to promote mitochondrial fusion [58]; syntaxin-17 (STX17), regulating mitochondrial fission with dynamin-related protein 1 (DRP1) [59]; and mitochondrial fission protein 1 (FIS1), an essential factor for mitochondrial fission, which forms a complex with B-cell-receptor-associated protein 31 (BAP31) in the MAMs [60]. Recently, a study reported that 16 of 21 ALS-causative genes alter the integrity of MAMs, compromising mitochondrial functions [61].

## 3. Secondary Mitochondrial Dysfunctions in ALS

The large number of genes and cellular processes associated with ALS suggests a multifactorial nature of the disease. Among the many mechanisms involved in the pathogenesis of ALS, mitochondria are thought to play an important role. Indeed, apart from their classical role as energy producers, mitochondria are also involved in other cellular functions that are impaired in ALS as described above. Several pathogenic variants in ALS genes, such as *SOD1*, *C9ORF72*, *TDP-43*, *FUS*, *ALSIN*, *VAPB*, *OPTN*, *SIGMAR1*, *SQSTM1* and *VCP*, are associated with impaired mitochondrial functions (Table 1).

### 3.1. SOD1/ALS1

Since the identification of the first ALS-causing mutations in the gene *SOD1* in 1993, important discoveries in the field of ALS have increased exponentially [62]. This gene, located on chromosome 21q22, encodes the enzyme Cu/Zn superoxide dismutase (SOD1). The SOD1 protein is an antioxidant that eliminates ROS in the cytosol and mitochondria [63,64]. Most *SOD1* mutations are pathogenic missense variants that are dominantly inherited. More than 200 mutations are known, the two most common being G85R and G93A. However, the clinical phenotype is highly variable, and patients with the same *SOD1* mutation show within-family differences in the severity of symptoms and speed of disease progression [65]. Mutations in *SOD1* can lead to toxic gain or loss of function, resulting in damage to cellular homeostasis. In ALS, *SOD1* mutations are reported to be involved in multiple mechanisms, such as protein degradation disorder, toxic protein aggregation, microglial inflammation, oxidative stress, oligodendrocytes and mitochondrial dysfunctions [66].

SOD1 protein is a cytosolic and mitochondrial antioxidant enzyme whose main function is the conversion of superoxide to molecular oxygen and hydrogen peroxide [63]. *SOD1* mutations have been reported to affect post-translational modifications, resulting in significant misfolding and aggregation of SOD1 protein [67]. Mutant SOD1 is frequently observed as aggregates at the OMM in MNs of various mouse models and in fALS patients [68]. These misfolded protein aggregates are hypothesized to lead to mitochondrial damage, including increased mitochondrial volume and excessive superoxide production. SOD1 aggregates located on the surface of the OMM lead to a decrease in mitochondrial membrane permeability. They directly inhibit respiration, increase oxidative stress and cause mitochondrial damage and cytochrome *c* release [69]. Misfolded SOD1 accumulates on the cytoplasmic surfaces of mitochondria by binding directly to VDAC [70]. Direct binding between SOD1 and VDAC inhibits conductance for adenine nucleotides and impairs energy supply to the MNs, leading to oxidative stress and consequent mitochondrial dysfunction [70]. Reduction of VDAC activity has been shown to decrease survival of SOD1 mutant mice. Mutant SOD1 can also bind directly to Bcl-2, a protein localized in the OMM that plays a role in promoting cell survival and inhibiting the action of pro-apoptotic proteins [71]. The interaction between SOD1 and Bcl-2 triggers a conformational change in Bcl-2 that exposes its toxic BH3 domain and converts Bcl-2 into a toxic protein, resulting in mitochondrial conformational changes and compromising the integrity of the mitochondrial membrane, which in turn results in the release of cytochrome *c* [71]. Mutant SOD1 damages ER–mitochondrial signaling through a loss of interaction between the Sigma1 receptor and the IP3 receptor, leading to dysregulation of Ca^2+^ homeostasis [72]. Mutant SOD1 aggregates also impair mitophagy by binding and sequestering optineurin (OPTN), an autophagy receptor required for mitophagosome formation, resulting in inhibition of OPTN translocation to mitochondria and accumulation of ROS, leading to dysfunctional mitochondria [73].

### 3.2. C9ORF72/ALS-FTD1

*Chromosome 9 open reading frame 72* (*C9ORF72*) is located on chromosome 9p21. *C9ORF72* encodes a protein of the same name that is mainly expressed in neurons. In 2011, two independent groups published results on non-coding hexanucleotide repeat expansion (HRE) of *C9ORF72* in ALS and ALS/FTD patients, establishing the first molecular link between these two diseases [74,75]. The mutations involve the expansion of an intronic hexanucleotide repeat (GGGGCC or G4C2) in the first intron of the *C9ORF72* gene. The hexanucleotide expansion is translated into dipeptide repeat (DPR) proteins, some of which have been shown to be neurotoxic [76,77,78]. Healthy individuals show less than 30 repeats, while ALS patients show hundreds and thousands of repeats (a small proportion of patients show expansion of 70 to 100 repeats) [79]. The *C9ORF72* expansion is present in up to 40% of fALS and up to 7% of sALS patients and is the most common known genetic cause of ALS and ALS/FTD. Patients are heterozygous for repeat expansion, and the mutation is dominantly transmitted. The pathogenic mechanisms by which repeat expansions in the *C9ORF72* gene cause ALS are not fully understood. Three distinct but not mutually exclusive disease mechanisms with loss-of-function or gain-of-function effects have been proposed [80]. Loss-of-function effects are associated with functional *C9ORF72* haploinsufficiency. Gain-of-function effects, on the other hand, are due to sequestration of RNA-binding proteins (RNPS) by RNA foci containing the *C9ORF72* gene HRE RNA and dipeptide repeat protein (DPR) production by a process called repeat-associated non-AUG (RAN) translation. The expanded RNA molecules can be translated into various DPR that form protein aggregates in the brain and spinal cord of *C9ORF72*-ALS/FTD patients [81,82]. C9ORF72 aggregation leads to TDP-43 proteinopathy [83].

The association between C9ORF72 and mitochondrial dysfunction was first demonstrated in 2016 with increased oxygen consumption, mitochondrial hyperpolarization, increased ROS and increased ATP content in *C9ORF72* fibroblasts [84]. The arginine-containing poly(GR) may act as a mitochondrial targeting signal, causing a portion of the poly(GR) to be translationally imported into mitochondria. Poly(GR) translation occurs on the mitochondrial surface and is frequently stalled, triggering ribosome-associated quality control and C-terminal expansion that promotes poly(GR) aggregation on mitochondria [85] and impairs mitochondrial functions and causes increased oxidative stress and DNA damage [86]. Poly(GR) expression alters mitochondrial membrane potential, increases DRP1 and decreases OPA1 protein expression and impairs complex I and ATP synthase activity by promoting degradation of ATP synthase F1 subunit α (ATP5A1) [87]. Dysfunction in OXPHOS complexes I and IV was reported with an increase in ROS levels in the *C9ORF72* mutant models [88]. The decrease in complex I activity may be explained by a decrease in mutant C9ORF72 protein levels, because C9ORF72 regulates OXPHOS by preventing the degradation of the translocase of inner mitochondrial membrane domain-containing 1 (TIMMDC1), which is fundamental for OXPHOS complex I assembly [89]. The C9ORF72 mutant MNs exhibit shorter axons, impaired rapid axonal transport of mitochondrial cargo and altered mitochondrial bioenergetic function [90]. A decrease in C9ORF72 protein was observed in ALS patients and iPSC-derived MNs [91,92]. This reduced C9ORF72 expression may alter mitophagy initiation and potential lysosomal degradation [93,94]. Mutant *C9ORF72* leads to disruption of ER-mitochondria contacts and Ca^2+^ exchange via an effect on the VAPB-PTPIP51 tethers [56,95,96].

### 3.3. TDP-43/ALS10

The 43 KDa trans-activating response region DNA-binding protein 43 (TARDBP or TDP-43), encoded by the *TARDBP* gene on chromosome 1p36, belongs to the heterogeneous nuclear ribonucleoprotein family, a group of proteins involved in RNA processing [97]. It is abundantly expressed in most organs, such as the central nervous system. This RNA-binding protein contains two RNA-recognition motifs (RRMs), a nuclear localization signal (NLS) and a nuclear export signal (NES), which are critical for intracellular localization. A *TDP-43* mutation was first reported in 2007 in a Japanese family in which four members in two generations developed adult ALS, with relatively rapid progression of bulbar symptoms [98,99]. The majority of *TDP-43* mutations linked to ALS and FTD are in the C-terminal glycine-rich region that mediates protein–protein interactions [100], although some are also found in the two highly conserved RRMs [101,102]. Most *TDP-43* mutations are missense mutations, such as D169G, G298S, A315T, Q331K, M337V, Q343R, A382T, etc., for which several ALS-disease models have been established [103].

In 2006, TDP-43 was identified as the major component of the insoluble and ubiquitylated aggregates observed in the MNs of ALS and ALS/FTD patients [14,15]. Under physiological conditions, TDP-43 is mainly localized in the nucleus, where it regulates mRNA expression and splicing. Following an export signal from the nucleus, TDP-43 migrates to the cytoplasm to regulate the stability, transport and translation of specific mRNAs along axons and dendrites. However, under stress conditions, a hyperphosphorylated, ubiquitinated and cleaved form of TDP-43 aggregates in the cytoplasm, leading to axonal swelling and impaired mobility [14,104]. Abnormal processing and aggregation of TDP-43 is usually defined as TDP-43 proteinopathy. It has been reported that more than 90% of sALS patients express cytoplasmic aggregates of TDP-43 [105]. TDP-43 inclusions in ALS/FTD are also hyperphosphorylated, ubiquitinated and C-terminally truncated as seen in ALS [14]. Although the pathogenic role of TDP-43 in the development of ALS is well established, the role of TDP-43 as a biomarker of ALS remains controversial.

TDP-43 proteinopathy leads to oxidative stress and dysfunction of mitochondria, contributing to the pathogenesis of ALS. Several animal models in which wild-type TDP-43 is overexpressed result in neurodegeneration similar to that observed in ALS, accumulate mitochondria in cytoplasmic inclusions in MNs, lack mitochondria in motor axon terminals or exhibit abnormal juxtanuclear aggregates of mitochondria in spinal MNs, suggesting that wild-type TDP-43 impairs mitochondrial function [31,106,107]. TDP-43 colocalizes with mitochondria in MNs, and ALS-associated mutants enhance its localization with mitochondria [108]. Mitochondrial localization of TDP-43 depends on three internal motifs: M1 (aa 35–41), M3 (aa 146–150) and M5 (aa 294–300) [109]. This mitochondrial localization is linked with dysfunction in mitochondrial dynamics and morphology and a reduction in mitochondrial length and density resulting from interactions between TDP-43 and the mitophagy regulatory protein prohibitin 2 and the fusion protein MFN2 [110]. Mitochondrial localization of TDP-43 appears to be central to its toxic effects on mitochondria. Indeed, a peptide that specifically inhibits mitochondrial TDP-43 addressing without affecting the localization and molecular functions of nuclear or cytoplasmic TDP-43 restores mitochondrial function, ameliorates neurodegenerative damage and enhances motor performance in several TDP-43-mutated mouse models of ALS [111]. Mitochondrial TDP-43 accumulation triggers the release of cytosolic mtDNA via the permeability transition pore (mPTP), leading to stimulation of the cGAS/STING pathway. STING may trigger activation of NF-kB and type I IFN pathways, both of which are elevated in ALS and contribute neurodegeneration progression [112]. TDP-43 mislocation and aggregation is associated with quality control defects in mitochondria, which may disturb the ER–mitochondria contacts and prevent protein import to the mitochondrial matrix [113]. TDP-43 mutants disrupt the interaction between VAPB and PTPIP51 and cellular Ca^2+^ homeostasis, disturbing interactions between mitochondria and ER, which are involved in various physiological processes, such as ATP production, mitochondrial biogenesis and apoptosis [95]. Mutant TDP-43 impairs electron transfer complex I assembly by binding to mitochondrial RNA (mtRNA) transcripts encoding respiratory complex I subunits NADH-ubiquinone oxidoreductase chains 3 and 6 (ND3 and ND6) [109]. TDP-43 plays a role in maintaining mitochondrial homeostasis by regulating mitochondrial transcript processing through direct binding to mitochondrial mtDNA-derived transcripts [114].

### 3.4. FUS/ALS6

Fused in sarcoma (FUS), encoded by the *FUS* gene on chromosome 16p11, is one of the RNA-binding proteins. FUS is ubiquitously expressed and plays an important role in DNA repair and RNA metabolism, including transcription, splicing, mRNA transport and mRNA translation [115,116]. Under physiological conditions, FUS is mainly localized in the nuclear compartment and undergoes nucleocytoplasmic shuttling. In the cytoplasm, FUS is found in RNA granules and diffusely in non-RNA granules in axons, dendrites and synapses. *FUS* mutations were first identified in 2009 in ALS patients, but none of the affected individuals developed cognitive deficits. Cytoplasmic FUS aggregates in spinal cord motor neurons were also observed in the motor cortex [117]. More than 50 different mutations in the *FUS* gene have been identified, causing up to 4% of fALS and 1% of sALS disorders [118,119]. The FUS-related ALS phenotype is characterized by young age and aggressive disease progression [120,121], with bulbar and spinal forms. Few *FUS* mutations have been identified in familial FTD [122]. Most *FUS* mutations are missense mutations found predominantly in the 3′-arginine/glycine-rich regions and the nuclear localization signal (NLS) of the protein. They cause cytoplasmic mislocalization of FUS and lead to pathological aggregation of FUS protein, which is thought to be responsible for neuronal degeneration in ALS [123]. The mechanisms associated with *FUS* mutations are described as loss of function in the nucleus and toxic gain of function in the cytoplasm [124,125]. FUS shares many functional and pathological mechanisms with TDP-43, but TDP-43 aggregates are not present in FUS-ALS patients.

In *Fus* mouse and drosophila models, NMJ degeneration is associated with mitochondrial abnormalities in the early presymptomatic phase of the disease [126,127]. FUS protein may interact with the mitochondrial chaperone heat shock protein (HSP60) in mitochondria, and the translocation of FUS to mitochondria is mediated, at least in part, by HSP60. Downregulation of HSP60 reduces FUS localized to mitochondria and partially rescues mitochondrial defects, such as increased ROS levels, and neurodegenerative defects caused by FUS expression in transgenic flies [128]. FUS is also involved in synaptic and mitochondrial function through its interaction with the mitochondrial anchoring protein syntaphilin (SNPH), which is essential for synapse maintenance [129]. Disruption of ER–mitochondrial interactions has been reported in FUS-related ALS caused by activation of glycogen synthase kinase-3β, leading to disruption of the VAPB-PTPIP51 interaction. This disruption is associated with impaired Ca^2+^ uptake by mitochondria following its release from the ER [96]. FUS is also associated with fragmented mitochondria and impaired mitochondrial ATP production [96]. FUS directly regulates the expression of Parkin [130], and loss of Parkin can impair mitophagy. Mutant FUS also impairs axonal transport in mitochondria by regulating the expression of several kinesins [131]. FUS associates with several mRNAs encoding sub-units of the mitochondrial respiratory chain, causing mitochondrial dysfunction [132]. This sequestration results in decreased levels of OXPHOS proteins sufficient to cause disorganized mitochondrial networks and increased ROS.

### 3.5. Alsin/ALS2

The *ALS2* gene, located on chromosome 2q33 in humans, encodes alsin, a 184 kDa protein [133,134]. ALS2 is ubiquitously and abundantly expressed in various tissues, including MNs. Approximatively 100 pathogenic variants in *ALS2* have been identified in patients with MNDs [135,136] and cause a number of autosomal recessive juvenile-onset MNDs, including ALS type 2 (ALS2), infantile-onset ascending hereditary spastic paraplegia (IAHSP) and juvenile primary lateral sclerosis (JPLS) [133,134,137]. These MNDs are characterized by selective and progressive degeneration of the MNs, particularly the upper MNs. In juvenile ALS, the disease occurs between the ages of 3 and 20 years. All affected individuals present with a plastic pseudobulbar syndrome (spasticity in speech and swallowing) along with spastic paraplegia. Peroneal muscular atrophy is observed in some (not all) affected individuals. The disease is characterized by progressive spasticity of the limbs and facial and pharyngeal muscles with spastic gait and spastic dysarthria. Some patients have amyotrophy of the hands and peroneal muscles. Several patients present with pseudobulbar symptoms [138,139].

The encoded protein alsin belongs to the guanine nucleotide exchange factors (GEFs) and activates the small GTPase Rab5 [140]. The presence of several GEF domains, including three major domains, the N-terminal regulator of chromosome condensation 1-like domain (RLD), the central Dbl homology and pleckstrin homology domain (DH/PH) and the C-terminal vacuolar protein sorting 9 domain (VPS9), suggests that ALS2 functions as a regulator/activator of several small GTPases [141,142]. The presence of these functional domains suggests important subcellular functions of alsin, including modulation of endosome and mitochondrial transport and endocytosis [140,143,144,145]. In alsin KO mice, corticospinal MNs exhibited vacuolated apical dendrites with increased autophagy, shrinkage of soma size and axonal pathology. They also exhibited abnormal mitochondria with defective inner mitochondrial membranes and damaged cristae [146]. The GTPase Rab5 translocates from endosomes to mitochondria upon oxidative stress. This process is reversible and is accompanied by an increase in Rab5-positive endosomes in contact with mitochondria. Alsin-deficient human pluripotent stem-cell-derived spinal MNs are defective in translocating Rab5 to mitochondria and show increased susceptibility to oxidative stress. These results suggest that alsin catalyzes the assembly of the endocytic Rab5 machinery onto mitochondria. Defects in stress recognition by endosomes may be critical for mitochondrial quality control during the onset of ALS [145].

### 3.6. VAPB/ALS8

The *VAPB* gene, located on chromosome 20q13 in humans, encodes a 27 kDa protein that belongs to the vesicle-associated membrane-protein (VAMP)-associated protein (VAP) family. In 2004, a missense mutation in the *VAPB* gene (p.P56S) was described in a large Brazilian family [147]. Patients presented clinical and neurological symptoms consistent with a diagnosis of ALS with slow progression. Clinical symptoms occurred between the ages of 31 and 45 years. All patients had lower MN symptoms and some have bulbar involvement.

VAPB is an integral protein of ER and promotes unfolded protein response (UPR), a process that suppresses the accumulation of unfolded proteins in the ER [148]. VAPB is highly expressed in the MNs of the spinal cord [149]. The interaction between VAPB and PTPIP51 in MAMs [150] is crucial for a number of key cellular functions, including IP3R-mediated calcium homeostasis, lipid synthesis, autophagy and synaptic activity in neurons [56,95,150,151,152]. Expression of the P56S mutation in neurons selectively disrupts anterograde mitochondrial axonal transport by disrupting Ca^2+^ homeostasis and impairing Miro1/kinesin-1 interaction with tubulin [153]. Interestingly, overexpression of VAPB in the SOD1G93A transgenic mouse model delayed the ALS phenotype and prolonged the lifespan of the mice by three and a half days [154].

### 3.7. OPTN/ALS12

The *OPTN* gene, located on chromosome 10p13 in humans, encodes optineurin, a 64 kDa hexameric protein involved in mitophagy. It also plays a role in inflammation, vesicle transport from the Golgi to the plasma membrane and necroptosis [155]. Optineurin is ubiquitously expressed in all human organs including skeletal muscle and brain [156]. Mutations in *OPTN* are associated with both ALS and glaucoma. More than 20 mutations in *OPTN* are known, and autosomal dominant and autosomal recessive inheritance patterns have been reported. Two variants in this gene, the E478G and Q398X, were first identified in six Japanese individuals from consanguineous marriages who had ALS [157]. In all patients with *OPTN* mutations, the disease appeared between the ages of 30 and 60 years. Most of them showed relatively slow progression and long duration to respiratory failure, although clinical phenotypes were not homogeneous. Rare patients may also develop FTD [158,159]. ALS-associated loss-of-function mutations, including exon deletions, frameshifts and nonsense and missense mutations account for up to 4% of fALS and less than 1% of sALS [157,160].

Optineurin is an autophagy receptor in Parkin-mediated mitophagy [161], but it is not required for the execution of PINK/Parkin-independent mitophagy. Optineurin has a central role in Parkin-mediated mitophagy. Optineurin is recruited to damaged mitochondria by binding to ubiquitinated mitochondrial proteins and interacting with LC3 to link damaged mitochondria to autophagosome formation [162]. Its role in clearing damaged mitochondria is regulated through phosphorylation of OPTN by TANK-binding kinase 1 (TBK1) [163]. Optineurin recruits TBK1 to poly-ubiquitinated mitochondria, where TBK1 in turn phosphorylates optineurin, enhancing its binding to ubiquitin and facilitating LC3 recruitment and mitophagy [163]. Interestingly, optineurin and p62, which also plays a role in mitophagy, localize independently to different mitochondrial sites. p62 is not required for optineurin/LC3 recruitment to damaged mitochondria in HeLa cells, suggesting that optineurin and p62 regulate mitophagy by different mechanisms [162]. Nevertheless, TBK1 phosphorylates both optineurin and p62 during mitophagy [164]. ALS-associated *OPTN* mutants are a causative factor of mitochondrial dysfunction through loss of function, leading to ALS pathogenesis [162]. In some familial and sporadic ALS patients, optineurin proteins formed protein inclusions [157]. Optineurin was sequestered by SOD1 mutant leading to impaired mitophagy, causing an accumulation of damaged mitochondria, leading to cell death [73].

### 3.8. SIGMAR1/ALS16

The *SIGMAR1* gene, located on chromosome 9p13 in humans, encodes the Sigma-1 receptor (Sigma1R), a chaperone protein that is ubiquitously expressed in both the central and peripheral nervous systems [165,166]. It is enriched in MNs of the brainstem and spinal cord and plays a role in a variety of cellular functions critical for neuron survival and maintenance [167]. A pathogenic mutation in *SIGMAR1* has been identified in patients with FTD associated with MNDs resembling ALS [168]. In 2011, a homozygous pathogenic E102Q mutation in a conserved transmembrane region of Sigma1R was identified in affected members of a consanguineous Saudi Arabian family. This mutation is responsible for a juvenile autosomal recessive form of ALS [169].

SIGMAR1 is specifically localized in the MAMs. SIGMAR1 is involved in the transport of lipids such as cholesterol and galactoceramide and acts as a chaperone protein for IP3Rs to facilitate the transport of calcium into mitochondria [165,170]. Disruption of SIGMAR1 function in MNs results in MAM loss, impaired calcium homeostasis, ER stress activation and defects in mitochondrial dynamics and transport. Interestingly, inhibition of mitochondrial fission was sufficient to induce mitochondrial axonal transport defects and rescue axonal degeneration [170].

### 3.9. SQSTM1/ALS-FTD3

The *SQSTM1* gene, located on chromosome 5q35 in humans, encodes a 48 kDa (observed at 62 kDa) ubiquitin-binding protein called p62 or sequestosome 1. Heterozygous *SQSTM1* mutations were first reported in 2011 in familial and sporadic patients with ALS [171] and later identified in several ALS/FTD patients [172,173,174,175,176]. The phenotype is highly variable, even within a family. Patients present in adulthood or late adulthood with cognitive impairment, behavioral abnormalities, speech ataxia and/or upper and lower MN signs. Some patients may also develop Paget’s bone disease. Mutations in *SQSTM1* are associated with approximately 2% of fALS and 4% of sALS cases [172,177]. The p62 protein has previously been linked to MNDs through its localization in ubiquitin-positive cytoplasmic aggregates [178]. p62 is a key component in pathological aggregates observed in neurons in ALS and FTD patients [179].

SQSTM1/p62 is localized to mitochondria and is essential in genome integrity, regulation of mitochondrial morphology and mitochondrial import of a key transcription factor [180]. p62 seems implied in the protection of cells from oxidative damage and promotes cell survival. Defects in p62 result in oxidative damage and are associated with various neurodegenerative diseases [181]. Deficiency in p62 exacerbates defects in mitochondrial membrane potential and energetics leading to mitochondrial dysfunction. p62 is also involved in the regulation of TFAM, the mitochondrial transcription factor, and mitochondrial dynamics [180]. Continuous structural remodeling through fusion and fission is essential for efficient mitochondrial function and renewal [182]. p62 deficiency decreases mitochondrial potential membrane, inhibits mitochondrial respiration and increases cytosolic ROS production in vitro [183]. p62 is also a mitophagy receptor which associates directly to LC3 to enable the elimination of the mitochondria. It is recruited to depolarized mitochondria in PINK1/parkin-expressing cells [184]. The L341V mutation in the *SQSTM1* gene is defective in recognition of LC3B and thus impairs mitochondrial clearance [185]. The mitophagy process is dependent on the recruitment of both p62 and the ubiquitin-binding HDAC6 [184,186]. Although there are conflicting reports concerning the role of p62 in mitophagy, it is established that p62 is important in the polymerization and the transport of mitochondria to aggregates [187].

### 3.10. VCP/ALS14

The *VCP* gene, located on chromosome 9p13 in humans, encodes vasolin-containing protein (also called p97), an AAA-ATPase, part of the large family of ATPases associated with diverse cellular activities (AAA). VCP is abundantly expressed in several tissues, including brain and skeletal muscle. Several mutations have been reported in the *VCP* gene [188]. Many of them are located in exon 5 within the N-terminal CDC48 domain involved in ubiquitin binding, implying that mutations in this region may negatively affect the ubiquitin protein degradation pathway [189]. Mutations in *VCP* are associated with ALS/FTD *continuum* [190], pure ALS [191], Paget’s bone disease (PBD) [188], hereditary spastic paraplegia [192], Charcot–Marie–Tooth disease type 2 [193] and multiple dystrophic syndromes [194]. *VCP* mutations were first described in 2010 in an ALS Italian family. Affected individuals presented in adulthood (between 37 and 53 years of age) with symptoms that appeared in the limbs and progressed rapidly, affecting all four limbs and the bulbar musculature, consistent with a classic ALS phenotype. Several patients had FTD and one had Paget’s bone disease followed by ALS, suggesting an overlap between these two pathologies [191]. *VCP* mutations account for only 1–2% of fALS and have a small role in sALS [195]. Mutations in *VCP* lead to dysregulation of protein homeostasis with protein aggregation and accumulation, particularly of TDP-43 and tau protein [196,197].

VCP uses the energy provided by the hydrolysis of ATP to alter the conformations of target proteins and has a key role in cellular function maintenance and calcium homeostasis under physiological conditions, such as in the ubiquitin–proteasome system (UPS), in the delivery of misfolded proteins from the ER, in lysosomal homeostasis, in cell cycle regulation, in DNA damage response and in autophagy and mitophagy processes [198,199,200]. Impairment of VCP activity due to mutation or to lack of expression of the structure leads to ER and mitochondrial dysfunction, which subsequently causes cellular damage and leads to the development of the disease. VCP is essential in maintaining the integrity of the ER by interacting with E3 ubiquitin ligases, such as glycoprotein 78 (gp78) and the ERAD-associated E3 ubiquitin-protein ligase HRD1 (Hrd1) [201,202]. VCP is implied in removing the misfolded proteins from mitochondria during the mitochondria-associated degradation process with the VCP cofactors Ufd1-Np14. VCP is involved in mitophagy, depending on the E3 ligase Parkin [203]. And, it is implied in the calcium homeostasis regulation by MAMs through electron activity and ATP production. VCP prevents excessive calcium entry into mitochondria through the regulation of mitochondrial calcium uptake. This process is dependent of the degradation of mitochondrial calcium uptake proteins, which then inhibit the opening of the mPTP, thus preventing cell death [204]. In addition, VCP maintains function of mitochondria in neuronal cells through UPS and outer mitochondrial membrane-related degradation [205]. Recruitment of VCP to mitochondria requires the PINK1/Parkin pathway through ubiquitination of mitochondrial targets (such as the mitofusins Mfn1 and Mfn2) by Parkin [206]. In addition, VCP and its adaptor Npl4/Ufd1 are required for removal of damaged mitochondria via the PINK1/Parkin pathway. In ALS, *VCP* variants interfere with the removal of damaged mitochondria by impairing the PINK1/Parkin pathway [206]. Impairment of VCP activity leads to damage in protein clearance, autophagy, maintenance of lysosomal homeostasis and mitochondrial quality control, resulting in disease pathogenesis.

## 4. Primary Mitochondrial Dysfunction in *CHCHD10*-Related ALS

### 4.1. CHCHD10/ALS-FTD2

The *CHCHD10* gene, on chromosome 22q11, encodes Coiled-coil-Helix-Coiled-coil-Helix-Domain-containing 10 (CHCHD10), a 14 kDa mitochondrial protein belonging to the Coiled-coil-Helix (CHCH) domain family characterized by conserved CX9C motifs. These CX9C motifs are required for their import into mitochondria via the MIA40 pathway [207]. The first heterozygous variant of *CHCHD10* (p.Ser59Leu, S59L) was identified in two families in 2014. In the first, a large, French family, patients showed mitochondrial myopathy with mtDNA instability, motor neuron damage (ALS-like) and cognitive decline resembling FTD [22]. In the second family, the same variant was responsible for a classic form of ALS/FTD [22]. The inheritance pattern was autosomal dominant with complete penetrance. In muscle, affected members exhibited deletions of mitochondrial DNA (mtDNA), COX-negative and ragged red fibers [22]. It was the first genetic evidence that a mutation of a gene encoding a mitochondrial protein can trigger MND.

*CHCHD10* mutations were identified in both fALS and sALS [23,24,208,209,210,211,212,213,214], in ALS/FTD [22,215,216] and in pure FTD [211,215,217,218,219]. Variants have also been reported in cohorts with Charcot–Marie–Tooth Type 2 (CMT2) [220] and spinal muscular atrophy, Jokela type (SMAJ) [221,222,223,224]. Variants have also been described in mitochondrial myopathies and cardiomyopathies [219,225,226,227]. Mutations in *CHCHD10* lead to toxic gain of function, resulting in overall mitochondrial dysfunction [228,229]. Since 2014, more than twenty *CHCHD10* missense mutations have been identified, but only four have been studied in depth: R15L [23,24,230], G58R [225,231], S59L [22,216] and G66V [24,222,223]. Interestingly, the patients developed different clinical phenotypes. All these results indicate that *CHCHD10* is involved in a broad spectrum of diseases that share an initial mitochondrial defect. It was hypothesized that different mechanisms might be involved in *CHCHD10* pathogenesis, differing for each variant.

### 4.2. CHCHD10 and Mitochondrial Impairments in ALS/FTD

**Figure 2:**
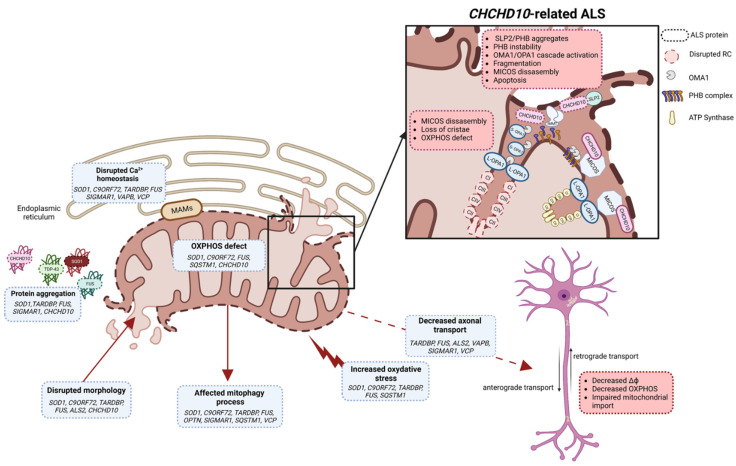
Schematic illustration of different mitochondrial functions impacted by ALS variants (created with biorender.com, accessed on 29 September 2023). Several mechanisms leading to mitochondrial dysfunctions, such as protein aggregation, OXPHOS, oxidative stress, Ca^2+^ homeostasis, mitochondrial morphology, mitophagy and axonal transport, have been described in ALS. In motor neurons, mitochondrial dysfunction leads to decreased mitochondrial membrane potential, decreased OXPHOS and impaired mitochondrial import. In *CHCHD10*-related ALS, SLP2/PHB aggregates and PHB complex instability are key factors which trigger the OMA1/OPA1 cascade leading to the imbalance in S-OPA1/L-OPA1 forms, abnormal mitochondrial dynamics and apoptosis. PHB instability and disrupted OPA1-mitofilin interaction should lead to instability of MICOS complex. CHCHD10 is also a partner of MICOS, and in ALS it participates in MICOS complex disassembly, loss of cristae structure and OXPHOS defects. MAMs: mitochondrial-associated membranes; MICOS: mitochondrial contact site and cristae organizing system; RC: respiratory chain; IMMT: mitofilin; S-OPA1: short form of OPA1; L-OPA1: long form of OPA1; ΔФ: mitochondrial membrane potential; PHB: prohibitin complex.

CHCHD10 is localized in the intermembrane space of mitochondria and enriched at cristae junctions [22]. CHCHD10 is associated with the mitochondrial contact site and cristae organizing system (MICOS) complex through its interaction with the core protein mitofilin [232]. The MICOS complex is essential for cristae structural organization and OXPHOS functions. Mutation in *CHCHD10* leads to disorganization of the MICOS complex, decrease in mtDNA repair capacity of cells under oxidative stress and altered mitochondrial morphology with loss of cristae junctions and OXPHOS functions [232,233] (Figure 2).

CHCHD10 has also been shown to interact with the IMM stomatin-like protein 2 (SLP2) and participates in the stability of the prohibitin (PHB) complex in the IMM [233]. In the *Chchd10^S59L/+^* mouse model, SLP2 forms aggregates with prohibitins in the hippocampus and aggresome-like inclusions in spinal motor neurons. Affected cells and tissues display instability of the PHB complex [233]. All these results indicate a key role of CHCHD10 in the ultrastructural stabilization of the IMM. The integrity of the IMM is monitored by quality control mechanisms that include the protease OMA1 [234,235]. Under stress conditions for mitochondria, OMA1 is activated and inhibits the fusion of dysfunctional mitochondria by cleaving the fusion protein OPA1 [236]. Instability of the PHB complex has been shown to be involved at least in part, in activation of the OMA1 cascade, with OPA1 processing leading to mitochondrial fragmentation, abnormal mitochondrial cristae morphogenesis and neuronal death [233]. Destabilization of the PHB complex should lead to instability of the MICOS complex by disrupting the OPA1-mitofilin interaction [233] (Figure 2). Pathogenic mutations of *CHCHD10* lead to an increase in the mitochondrial integrated stress response (mt-ISR), resulting in activation of OMA1 [227]. Activation of the mt-ISR and metabolic rewiring have also been shown to be involved in mitochondrial dysfunction and pathogenesis of the S59L variant, suggesting a role for stress as a pathological mechanism for this variant [237].

In mitochondria, CHCHD10 can associate with its paralog CHCHD2 [238,239]. CHCHD2 is associated with PINK1/Parkin cell death and neurodegenerative diseases such as Parkinson’s disease [240,241], FTD [242], Alzheimer’s disease [242] and Lewy-body dementia [243]. The CHCHD10/CHCHD2 heterodimers are normally unstable, but in the presence of the S59L variant they aggregate, which can lead to a toxic gain of function [244]. Loss of CHCHD10, CHCHD2 or both affects mitochondrial respiration [239,245], mt-ISR [229,237,246,247,248], apoptosis [232,249] and transcription of the oxygen-responsive gene *COX4I2* [250,251,252], suggesting involvement in OXPHOS regulation (Figure 2).

*CHCHD10* mutants were found to aggregate, which has been identified as one of the pathological mechanisms linked to ALS pathogenesis. CHCHD10 mutants are associated with TDP-43 proteinopathy in several cell and animal models [29,228,229,253,254]. The R15L and S59L mutations have been shown to trigger CHCHD10 and TDP-43 proteinopathies that originate in mitochondria and directly reflect functional changes in long-term synaptic plasticity and motor unit physiology [255]. In contrast, CHCHD10 wild-type attenuates TDP-43 proteinopathy and rescues TDP-43-induced impairment of long-term synaptic plasticity, suggesting that CHCHD10 variants directly regulate the stability and aggregation of CHCHD10 and TDP-43 in mitochondria [255] (Figure 2).

NMJ fragmentation was observed in a *Chchd10^S59L/+^* mouse model, and the onset of disease occurs in muscle before MNs are affected [29]. A growing number of studies suggest that NMJ degeneration plays a key role in the progression of ALS. A theory suggests that ALS may be due to an NMJ defect that provokes MN death, which then leads to muscle wasting; this is the concept of “dying back” [256]. The results obtained in the Chchd10 mouse mutant would suggest the “dying back” theory as one of the pathological mechanisms of CHCHD10 in ALS.

## 5. Current Drugs Used in ALS

A variety of experimental drugs have been shown to delay disease progression in ALS preclinical animal models but have not shown efficacy in human trials or are awaiting approval in phase I–III trials. Currently, there are four drugs that prolong survival by several months or slow functional decline: the glutamate antagonist Riluzole, the antioxidant Edaravone, the combination between Sodium phenylbutyrate and Taurursodiol, and the antisense oligonucleotide Tofersen (Table 2).

The first FDA (Food and Drug Administration) drug approved for clinical use in ALS in 1995 was Riluzole. Riluzole belongs to the class of benzothiazoles, a glutamate antagonist. The mechanism by which Riluzole improves survival is still unclear. Riluzole appears to have a neuroprotective effect by inhibiting glutamate reuptake in the MN synapses and inactivating voltage-dependent sodium channels (i.e., reducing hyperexcitability) [257]. The molecular and cellular mechanisms of Riluzole are related to inhibition of glutamate release, inhibition of protein kinase C and affecting intracellular events that follow transmitter binding to excitatory amino acid receptors [258,259]. In addition, mitochondrial dysfunction in MNs inhibits the complex IV of the electron transport chain, leading to the formation of ROS. In the context of increased ROS levels, Riluzole inhibits Ca^2+^ efflux at synapses and decreases mitochondrial membrane potential [260,261]. ROS formation is blocked by Riluzole in MNs by inducing glutathione synthesis [262]. In ALS patients, an improvement in bulbar and skeletal muscle (but not total muscle strength) was observed. The median survival time was prolonged by 2–3 months [257].

Edaravone (MCI-186, 3-methyl-1-phenyl-2-pyrazolin-5-one) was approved for clinical use in Japan in 2015 and by the FDA in 2017. It is a free radical scavenger originally developed by Mitsubishi Chemical Industries Ltd. (Tokyo, Japan) for the treatment of neuropathies caused by cerebral infarction [263]. Edaravone is thought to reduce the effects of oxidative stress in MNs and slow the decline of physical function [264,265,266]. A growing body of experimental and clinical evidence supports the neuroprotective effects of Edaravone as an antioxidant that removes oxygen radicals and eliminates lipid peroxides in the central nervous system [267,268]. The drug clinically slows disease progression and appears to be effective in patients at an early stage of the disease [257].

Sodium phenylbutyrate and Taurursodiol (PB/TURSO, AMX0035) were approved by the FDA in September 2022. PB/TURSO was conditionally approved in Canada in 2022 because it has shown significant slowing of disease progression and prolongation of survival (median survival time increased by 4.8 months (Center for Drug Evaluation and Research, AMX0035 clinical overview and statistical review)). PB/TURSO reduces neuronal cell death by decreasing ER stress and mitochondrial dysfunction [269,270,271,272,273].

Tofersen was approved by the FDA in April 2023 for the treatment of ALS in adults with a mutation in the *SOD1* gene [274]. It is an antisense oligonuleotide that degrades SOD1 messenger RNA and reduces SOD1 protein levels [63,275].

## 6. Conclusions

Despite the increasing number of genes associated with ALS, the pathogenesis of ALS is still largely unknown. Mitochondrial dysfunction has emerged as a common phenomenon in ALS. The presence of deficits in OXPHOS, calcium homeostasis and mitochondrial transport before the onset of disease symptoms suggests that mitochondrial dysfunction plays an important role in the pathogenesis of ALS. Nevertheless, clinical trials targeting mitochondria have been disappointing, suggesting that mitochondrial dysfunction alone may not be the primary cause of the disease. Mitochondrial damage in ALS was hypothesized to be a consequence and an exacerbating factor of the disease. The identification of *CHCHD10* variants in ALS and ALS/FTD patients was the first genetic evidence that a mitochondrial defect may be a primary cause of damage in MNs and directly links mitochondrial dysfunction to the pathogenesis of ALS and FTD.

The multiple pathogenic processes demonstrated in ALS pathogenesis suggest that multiple pathways converge to a common endpoint leading to loss of MNs. This may explain the disappointing results obtained when a single pathological process was targeted. Because neuronal loss in ALS is most likely due to a combination of multiple pathogenic mechanisms, including mitochondrial dysfunction, oxidative stress and disruption of axonal transport processes, a multidrug approach is likely required. In the last 20 years since Riluzole was first approved, more than 50 clinical trials have been conducted [276]. Several of these targeted mitochondrial dysfunction, such as coenzyme Q10 [277], but none increased mitochondrial function and survival or decreased oxidative stress. Improvements were obtained in animal models but not in clinical trials. Nonetheless, since 2017, two of the FDA-approved drugs used in ALS patients target mitochondrial function: Edaravone, an antioxidant that reduces the effects of oxidative stress in MNs, and the combination of Sodium phenylbutyrate and Taurursodiol (PB/TURSO), which reduces neuronal cell death by reducing ER stress and mitochondrial dysfunction. Although ALS is a disease with many pathological mechanisms, targeting mitochondrial dysfunction seems to be a promising avenue for developing therapies in this disease.

## Figures and Tables

**Table 1:** Mitochondrial dysfunctions associated with ALS genes.

		Gene	Name	Affected Mitochondrial Functions
**Mitochondrial dysfunctions in ALS**	**ALS1**	***SOD1***	*Cu/Zn superoxide dismutase 1*	Aggregates, increased oxidative stress, impaired RC activity, disrupted mitochonrial dynamics and morpholgy, disrupted mitophagy, disrupted ER-mitochondria contacts and calcium homestasis
	**ALS-FTD1**	***C9ORF72***	*Chromosome 9 open reading frame 72*	Increased oxygen consumption, mitochondrial hyperpolarization, impaired RC activity, disrupted mitophagy, disrupted mitochonrial dynamics and morphology, disrupted ER-mitochondria contacts
	**ALS10**	***TARDBP/TDP-43***	*Trans-activating response region DNA-binding protein 43*	Agreggates, accumulation in mitochondria, increased oxidative stress, disrupted mitochondrial dynamics and morphology, disrupted mitophagy, disrupted ER-mitochondria contacts and calcium homestasis
	**ALS6**	***FUS***	*Fused in Sarcoma*	Agreggates, increased oxydative stress, impaired ATP production, disrupted mitophagy, disrupted ER-mitochondria contacts and calcium homeostasis
	**ALS2**	***ALS2***	*Alsin*	Disrupted mitophagy, disrupted mitochondrial morphology, disrupted endosomal and mitochondrial transport
	**ALS8**	***VAPB***	*Vesicle-associated membrane protein-associated protein B*	Disrupted ER-mitochondria contacts and calcium homeostasis, disrupted anterograde axonal transport
	**ALS12**	***OPTN***	*Optineurin*	Inclusions, disrupted mitophagy
	**ALS16**	***SIGMAR1***	*Sigma-1 receptor*	Agreggates, disrupted mitochondrial dynamic, disrupted ER-mitochondria contacts and calcium homeostasiss, disrupted axonal transport
	**ALS-FTD3**	***SQSTM1***	*p62/Sequestosome 1*	Agreggates, increased oxidative stress, decreased mitochondrial respiration, disrupted mitophagy, disrupted mitochondrial membrane potential
	**ALS14**	***VCP***	*Valosin-containing protein*	Disrupted ER-mitochondria contacts and calcium homeostasis, disrupted mitophagy
	**ALS-FTD2**	***CHCHD10***	*Coiled-coil-helix-coiled-coil-helix domain containing 10*	Aggregates, disrupted mitochondrial dynamics and morphology, decreased mitochondrial respiration, disrupted mitochondrial potential, mt-ISR activation

**Table 2:** Current drugs used in ALS. FDA approved drugs: Riluzole, Edaravone, Sodium phenylbutyrate and Taurursodiol (PB/TURSO), and Tofersen. Two of them target mitochondrial functions (shown in red letters). ER: endoplasmic reticulum; FDA: Food and Drug Administration.

Name	FDA Approved (Year)	Mechanisms
***Riluzole***	1995	Glutamate antagonist that inhibits glutamate release and protein kinase *c*, regulates intracellular events that follow transmitter binding to excitatory receptors and regulates calcium flux
***Edaravone***	2017	Antioxidant that removes oxygen radicals and eliminates lipid peroxides in the central nervous system
***Sodium phenylbutyrate and Taurursodiol (PB/TURSO)***	2022	Decreases ER stress and mitochondrial dysfunction
***Tofersen***	2023	Antisense oligonucleotide that degrades SOD1 messenger RNA and reduces SOD1 protein levels

## Data Availability

The data presented in this study are available on request from the corresponding author.

## Abbreviations

ALS	amyotrophic lateral sclerosis
fALS	familial ALS
sALS	sporadic ALS
CHCHD10	Coiled-coil-Helix-Coiled-coil-Helix Domain containing 10
DRP1	dynamin-related protein 1
ER	endoplasmic reticulum
FIS1	fission protein 1
FTD	frontotemporal dementia
FUS	fused in sarcoma
IMM	inner mitochondrial membrane
IMS	intermembrane space
IP3	inositol 1,4,5-triphosphate
IP3R	inositol 1,4,5-triphosphate receptor
MAM	mitochondrial-associated membranes
MFN	mitofusin
MICOS	mitochondrial contact site and cristae organizing system
MN	motor neuron
MND	motor neuron disease
mtDNA	mitochondrial DNA
mt-ISR	mitochondrial integrated stress response
NMJ	neuromuscular junction
OMM	outer mitochondrial membrane
OPA1	optic atrophy 1
OPTN	optineurin
OXPHOS	oxidative phosphorylation
PHB	prohibitin
PTPIP51	protein tyrosine phosphatase interacting protein-51
ROS	reactive oxygen species
SLP2	stomatin-like protein 2
SOD1	superoxide dismutase 1
TARDBP/TDP-43	trans-activating response region DNA-binding protein 43
UPR	unfolded protein response
VAPB	vesicle-associated membrane protein-associated protein B
VCP	vasolin-containing protein
VDAC	voltage-dependent anion-selective channel proteins
